# Leech blood‐meal invertebrate‐derived DNA reveals differences in Bornean mammal diversity across habitats

**DOI:** 10.1111/mec.15724

**Published:** 2020-11-27

**Authors:** Rosie Drinkwater, Tommaso Jucker, Joshua H. T. Potter, Tom Swinfield, David A. Coomes, Eleanor M. Slade, M. Thomas P. Gilbert, Owen T. Lewis, Henry Bernard, Matthew J. Struebig, Elizabeth L. Clare, Stephen J. Rossiter

**Affiliations:** ^1^ School of Biological and Chemical Sciences Queen Mary University of London London UK; ^2^ School of Biological Sciences University of Bristol Bristol UK; ^3^ Department of Plant Sciences, Forest and Ecology Conservation Group University of Cambridge Cambridge UK; ^4^ Department of Zoology University of Oxford Oxford UK; ^5^ Asian School of the Environment Nanyang Technological University Singapore City Singapore; ^6^ Department of Biology University of Copenhagen Copenhagen Denmark; ^7^ University Museum NTNU Trondheim Norway; ^8^ Institute for Tropical Biology and Conservation Universiti Malaysia Sabah Sabah Malaysia; ^9^ Durrell Institute of Conservation and Ecology (DICE) School of Anthropology and Conservation University of Kent Canterbury UK

**Keywords:** biodiversity, Borneo, Haemadipsidae, invertebrate‐derived DNA, land‐use change, molecular biomonitoring

## Abstract

The application of metabarcoding to environmental and invertebrate‐derived DNA (eDNA and iDNA) is a new and increasingly applied method for monitoring biodiversity across a diverse range of habitats. This approach is particularly promising for sampling in the biodiverse humid tropics, where rapid land‐use change for agriculture means there is a growing need to understand the conservation value of the remaining mosaic and degraded landscapes. Here we use iDNA from blood‐feeding leeches (*Haemadipsa picta*) to assess differences in mammalian diversity across a gradient of forest degradation in Sabah, Malaysian Borneo. We screened 557 individual leeches for mammal DNA by targeting fragments of the 16S rRNA gene and detected 14 mammalian genera. We recorded lower mammal diversity in the most heavily degraded forest compared to higher quality twice logged forest. Although the accumulation curves of diversity estimates were comparable across these habitat types, diversity was higher in twice logged forest, with more taxa of conservation concern. In addition, our analysis revealed differences between the community recorded in the heavily logged forest and that of the twice logged forest. By revealing differences in mammal diversity across a human‐modified tropical landscape, our study demonstrates the value of iDNA as a noninvasive biomonitoring approach in conservation assessments.

## 1 INTRODUCTION

Tropical ecosystems are under pressure from deforestation (Hansen et al., 2013) and other anthropogenic activities driving forest degradation (Lewis et al., 2015). The removal of trees, and the associated damage from timber extraction, causes lasting changes to vegetation structure and microclimate, with knock‐on consequence for species diversity (Blonder et al., 2018). For example, microclimatic extremes are more frequent in logged forests than in older growth forests (Blonder et al., 2018; Hardwick et al., 2015; Jucker et al., 2018). In addition to altering floral and faunal community composition (Laurance et al., 2018; Wilkinson et al., 2018), logged forests can show changes in diverse ecosystem functions, including litter decomposition, predation and seed dispersal (Bovo et al., 2018; Robert M. Ewers et al., 2015). As a result, such forests show lower resilience to numerous local and climatic stressors (Struebig et al., 2015) and are at greater risk of conversion to commodity agriculture (Edwards et al., 2011).

Despite the well‐known negative effects of forest degradation on ecosystem processes, there is evidence that these degraded habitats can still support biodiversity and have considerably greater conservation value than alternative agricultural landscapes (Deere et al., 2017; Gibson et al., 2011). Even within highly degraded forest, animal community composition tends to be more similar to forest than it is to agricultural plantations (Gray et al., 2014; Wearn et al., 2017). Within heavily logged forest, for example, forest remnants have been shown to be important for birds (Mitchell et al., 2018) and invertebrates (Gray et al., 2014). These and other studies of how land‐use change relates to biodiversity have increasingly utilized data generated by LiDAR, an approach that allows new and improved opportunities to quantify forest structure and microclimatic variables across spatial scales (Asner et al., 2018; Deere et al., 2020; Seaman et al., 2019).

In recent years, the toolkit for biodiversity monitoring has expanded from solely field‐based methods to also encompass molecular techniques. In particular, advances in sequencing now allow for the routine metabarcoding of environmental DNA (eDNA) samples, thereby revolutionizing molecular ecology. One such area that has seen rapid progress is the use of animal‐feeding invertebrate species as samplers of vertebrate diversity. Invertebrate samplers have tended to be haematophagous species, of which arguably the most popular have been leeches (Abrams et al., 2019; Drinkwater et al., 2018; Fahmy et al., 2019; Schnell et al., 2018; Tessler et al., 2018; Weiskopf et al., 2017) and dipteran flies (Calvignac‐Spencer et al., 2013; Gogarten et al., 2019; Hoffmann et al., 2018; Kocher, de Thoisy, Catzeflis, Valiere, et al., 2017).

To date, invertebrate‐derived DNA (iDNA) has been widely utilized to obtain inventories of mammals and other vertebrate groups from tropical regions (e.g. Fahmy et al., 2019; Gogarten et al., 2019; Kocher, de Thoisy, Catzeflis, Valière, et al., 2017), but these have tended to focus on opportunistic invertebrate collection methods and comparisons of diversity across geographical regions (Schnell et al., 2018; Tessler et al., 2018). Yet because iDNA (and eDNA) can allow genetic confirmation of species presence without the need for actual observations, it can also complement more conventional monitoring based on, for example, camera trap surveys, with associated savings in fieldwork costs (e.g. Leempoel et al., 2020; Weiskopf et al., 2017). Abrams et al. (2019) have extended this further by analysing data from spatially matched iDNA and camera traps using an occupancy modelling framework and found that while both methods resulted in similar accumulation rates, the latter gave higher absolute species richness values. These authors also demonstrated that estimates of occupancy and detection probability varied depending on host species but also depended on leech type, with tiger leech (*Haemadipsa picta*) samples resulting in higher detection and occupancy probabilities compared to brown leeches (*Haemadipsa zeylanica*) (Abrams et al., 2019).

A further consideration of using iDNA is whether habitat has an effect on the efficacy of the invertebrate used for sampling (the so‐called “invertebrate sampler”). This may be particularly pertinent in the context of land‐use change, where small‐bodied invertebrates may be more sensitive to local conditions than the vertebrates for which they are being used to assay. It is not known, for example, whether local microclimate conditions will alter the foraging behaviour of invertebrate samplers, and thus their utility for comparing vertebrate diversity across habitats. Among the popular samplers are terrestrial leeches of the family Haemadipsidae, which are restricted to humid habitats and are adversely impacted by the drier conditions arising from forest degradation (Hardwick et al., 2015; Jucker et al., 2018). Previously we showed that forest structure affects the distributions of two congeneric haemadipsid leech species in logged forest (Drinkwater et al., 2019), yet it is not known whether such habitat preferences have additional implications for the detection of mammals.

Here we apply iDNA to assess the impact of habitat degradation on mammal diversity across a tropical landscape. Additionally, we test whether landscape‐scale variation in mammal diversity can be explained by temperature and humidity, which could suggest that detected patterns are mediated by leech responses to microclimate. To achieve this, we combined repeated surveys with a standardized collection protocol for tiger leeches across a degraded forest landscape in Malaysian Borneo. In this region, unsustainable logging practices coupled with land conversion for oil‐palm agriculture have led to the depletion of ancient dipterocarp forests, leaving behind managed landscapes that are fragmented and degraded (Gaveau et al., 2014).

## 2 MATERIALS AND METHODS

### 2.1 Study design and sample collection

**FIGURE 1:**
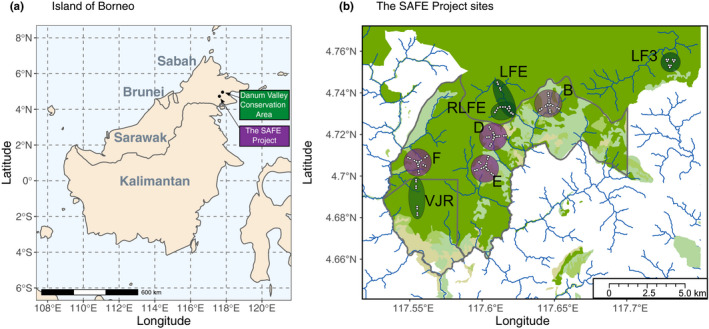
(a) Map of Borneo showing different regions, with the Danum Valley Conservation area and the SAFE project locality in Sabah marked by black circles. (b) SAFE study landscape showing the 25‐m^2^ plots (small white points) where leech samples were collected within the two habitat types: twice logged sites = LFE, LF3, RLFE and VJR (large green shading) and heavily logged sites = B, D, E and F (large purple shading)

Sampling was undertaken at The Stability of Altered Forest Ecosystems (SAFE) Project, Sabah, Borneo. This landscape has experienced varying degrees of logging disturbance since the 1970s and now comprises a mosaic of historically twice logged forest and more heavily degraded forest (Ewers et al., 2011). To understand the impact of this habitat degradation on mammal diversity through the use of iDNA, we analysed individuals of the terrestrial blood feeding tiger leech, *Haemadipsa picta*, collected at fixed locations across the SAFE landscape during a wet season between September and December 2016 (Table 1; Figure 1). The fixed locations were long‐term monitoring plots established by the SAFE project, which are grouped in larger sites, based on proximity (Ewers et al., 2011). We initially aimed to sample from plots in all of the 14 sites, excluding the sites established with oil palm plantations (OP1–3, Ewers et al., 2011). However, in reality only plots in eight sites could be sampled as a result of permit issues or access, such as poor roads. Repeated surveys were conducted within established 25‐m^2^ plots by searching the leaf litter and understorey for 20 min and collecting *H. picta* individuals and storing them in RNA Later (Qiagen) for subsequent molecular analyses (see Drinkwater et al., 2018). In addition, to extend habitat comparisons to pristine old growth forest, we also undertook equivalent surveys at the Danum Valley Conservation Area (DVCA), ~40 km away. Within each site, up to four repeat surveys were conducted at 8–12 plots over the season. Sites were classified into three levels of degradation based on logging history (Ewers et al., 2011): (a) old growth, (b) twice logged and (c) heavily logged, with the last experiencing recent salvage logging (for definitions of habitat type see Table S1).

**TABLE 1:** Site classification and sample sizes

Site	Habitat type	Individuals	Pools	Detections
OG	Old growth	114	12	16
VJR	Twice logged	27	3	5
LF3	Twice logged	99	10	23
LFE	Twice logged	130	13	22
RLFE	Twice logged	19	2	2
B	Heavily logged	70	7	12
D	Heavily logged	60	6	13
E	Heavily logged	28	3	5
F	Heavily logged	10	1	5

Note

At each site, we grouped individual leeches (3–11, mean = 9, median = 10) into pools prior to sequencing. Each site was then assigned a broad forest type classification and the number of mammal detections from each is given.

### 2.2 DNA extraction, PCR amplification and library pooling

To extract DNA, we incubated single whole leeches in lysis buffer and proteinase K overnight, following Drinkwater et al. (2018). After the initial digestion step, lysates from each leech were pooled by mixing 100 µl of site‐matched samples, specifically leeches collected from plots within the same site (Table S2 has full details of leech pools). To increase DNA yield we modified the DNA extraction protocol of Drinkwater et al. (2018) with the addition of an extra lysis step: 200 µl of buffer AL from DNeasy Blood and Tissue kit (Qiagen) to a 200‐µl subsample of each pooled digest which was then incubated for 15 min at 56°C. We then mixed in an additional 200 µl of 100% ethanol following the QiaQuick PCR purification protocol (Qiagen) but with reduced centrifuge speeds (6,000 g). For this study, we define a sample as a mix of iDNA from site‐matched leeches. Alongside each batch of the extractions we also conducted at least one extraction control (i.e., a blank sample that contained all of the reagents minus the tissue).

To minimize the risk of over‐inflation, leading to erroneously high diversity estimates, we used uniquely tagged, matching primers for each leech sample (Binladen et al., 2007) and polymerase chain reactions (PCRs) were conducted in triplicate. The primers we used were mammal‐specific with a target of a short (~95‐bp) fragment of the 16S rRNA gene (Taylor, 1996). To 1 µl of DNA template we added 0.2 mM of 10 × buffer, 2.5 mM MgCl_2_, 1 unit DNA polymerase (AmpliTaq Gold, Applied Biosystems), 0.2 mM dNTP mix (Invitrogen), 0.5 mg/ml bovine serum albumin, and 0.6 µm of the forward and reverse tagged primer, resulting in a final volume of 25 µl. The cycling profile was as follows (a) 95°C for 5 min, (b) 40 cycles of 95°C for 12 s, 59°C for 30 s and 70°C for 20 s and (c) a final extension time of 7 min at 70°C. Negative PCR and extraction controls were included in each batch of reactions and treated in the same way. Products were checked using 2% agarose gels and those reactions which showed amplification were mixed into amplicon pools (only containing unique tags) for a single‐tube library build (Carøe et al., 2017). The amplicon pools were sequenced in two batches, one at Queen Mary University of London's Genome Centre and the other at the Danish National High‐Throughput Sequencing Centre (University of Copenhagen), both with 150‐bp paired‐end chemistry with an Illumina MiSeq. The samples used in this study were multiplexed with other biological samples to increase complexity and thus accuracy of base calling.

### 2.3 Quality control, filtering and assigning sequences

Once sequenced, read pairs were merged using adapterremoval version 2 (Schubert et al., 2016). Data were demultiplexed based on nucleotide tag and library index combination using a modified version of dame and collapsed to unique sequences (https://github.com/shyamsg/DAMe, Zepeda Mendoza et al., 2016). To increase certainty in our assignments and to account for PCR stochasticity, we retained only unique sequences which appeared in a minimum of two out of the three PCR replicates. While this “relaxed restrictive” approach (Alberdi et al., 2017) lowers the overall detected diversity, it reduces bias and numbers of false positives from contamination and artefactual sequences. Next we filtered the sequences and only retained those sequences represented by more than 10 reads. To assign taxonomy we performed in silico PCR using the program ecopcr (Ficetola et al., 2010). To do this, we compared the 16S primer against all mammal sequences on GenBank (NCBI), allowing for a maximum of three mismatches between the query sequence and the primers. We generated an ecopcr database of all complementary sequences that could theoretically be amplified by our primer set. Using this database we mapped the unknown iDNA sequences using the *ecotag* command in the obitools package (Boyer et al., 2016) with a minimum identity of 0.95. We removed any sequence with an assignment above the genus level, as well as any assignment to a non‐native or geographically implausible mammal, and any human contaminant sequence. We only assigned sequences to a species for those mammal genera for which only one species representative is known to occur in Sabah. Within each leech pool, multiple assignments to the same taxon were collapsed, resulting in occurrence data or presence‐only, a common practice given the uncertainty of the link between sequence count and species abundance in the context of metabarcoding studies (Deagle et al., 2018; Elbrecht & Leese, 2015).

### 2.4 Accumulation of diversity

The successful detection of a mammal from leech‐ingested iDNA requires both that it was fed upon by the leech, and that its DNA is sufficiently intact for PCR amplification, and thus iDNA will propably underestimate actual diversity in a given habitat. Therefore, to estimate the alpha diversity of mammals based on the incidence of taxa we used the Chao2 estimators, which account for potential under sampling (Gotelli & Colwell, 2011). Diversity accumulation curves were generated for each forest type within a Hill number framework (Hill, 1973), using the “iNEXT” package in r (Hsieh et al., 2016) which uses the random acquisition of samples (Chao et al., 2014). Hill numbers are a way of unifying and generalizing the commonly used (but difficult to interpret) diversity indices, such as Shannon and Simpson indices, into more meaningful units, for example the effective number of species, or the number of equally abundant species needed to produce the same diversity value (Chao et al., 2014) or, for iDNA, equally abundant operational taxonomic units or OTUs (Alberdi & Gilbert, 2019). Different values of the scaling parameter, *q*, change the Hill number order of diversity based on sensitivity to rare species in the community. Thus, the most commonly used values, *q* = 0, 1, 2, are, respectively, equivalent to species richness, the exponential of the Shannon index and the inverse of the Simpson index (see Chao et al., 2014). The use of Hill numbers is recommended when incomplete sampling is expected (Chao et al., 2014). In iDNA‐derived estimates of diversity, additional sources of sample incompleteness can arise from the degradation of mammal DNA following digestion of the blood meal by the leech (Schnell et al., 2012), and the stochasticity of a PCR‐based amplification. Sample‐based accumulation curves were generated for each habitat type and for the three orders of diversity (*q* = 0, 1, 2), and curves were extrapolated to double the sample size of the observed value, following the maximum recommended extrapolation in Chao et al. (2014). Curves were plotted using 84% confidence intervals (CIs), which have been demonstrated to be equivalent to an alpha value of 0.05 when testing for significant differences between curves (MacGregor‐Fors & Payton, 2013; Payton et al., 2003). Research has shown that CIs can be overlapping by as much as half of one of the upper or lower intervals and still be equivalent to *p* = 0.05 (Cumming, 2009). Accumulation curves with the more traditional 95% interval are shown in Figure S1 for comparison. Using the more common rarefaction method, we calculated the diversity and sample coverage at each order of *q* (0, 1, 2) based on the smallest samples size with the *estimateD* function in iNEXT. These analyses were produced using “vegan” (Oksanen et al., 2017) and the “iNEXT” packages (Hsieh et al., 2016) in r (R Core Team, 2019) and figures were produced using “ggplot2” (Wickham, 2016) and “ggpubr” (Kassambara, 2020).

### 2.5 Community composition across a habitat gradient

We used nonmetric multidimensional scaling (NMDS) to visualize the community structure among sites of iDNA‐detected taxa. We used Chao's coefficients as our measure of dissimilarity between sites because this index accounts for the effect that undetected species have on the whole species pool and outperforms other dissimilarity indices when a large number of rare species are present in the sample (Chao et al., 2005). We ran the NMDS using the occurrence of taxa at each site for different numbers of axes (*k* = 2, 3, & 4) and assessed the resulting stress using screeplots. We present the NMDS with 95% CIs around the groups of sites within each habitat classification. To test for differences in variance between the habitat types, we used a permutational multivariate analysis of variance (PERMANOVA) All models were run for 9,999 permutations and constrained by site identity to reflect the study design. All NMDS and PERMANOVA analyses were conducted using the vegan package (Oksanen et al., 2017) and plots were generated using “ggordiplot” (Quensen, 2018), in r.

### 2.6 Microclimate variables

To investigate the effects of microclimate on the detection of mammals through leech iDNA, we considered four variables: maximum temperature, mean temperature, maximum vapour pressure deficit (VPD) and mean VPD, each of which was available for the entire SAFE landscape at a 50 × 50‐m resolution. These variables were generated for our key sites at SAFE by combining temperature and VPD measurements obtained from a landscape‐scale network of 120 microclimate dataloggers with high‐resolution maps of topography and canopy structure derived from airborne LiDAR (see Jucker et al., 2018, for full details). The four variables were extracted from the microclimate surface generated in Jucker et al. (2018), using the coordinates from the centre of each of the 25‐m^2^ plots. Values for Danum Valley Conservation Area (old growth forest) were extracted using the same approach, but from coordinates at fixed sites along rivers within the DVCA closest to the plots where we sampled. Values are shown in Figure S2 with points plotted using the function *geom_jitter* in the r package “ggplot2” to aid visualization on the horizontal axis.

### 2.7 Models of habitat type and microclimate on diversity

Because all four microclimate variables described (see above) covary, we conducted a principal component analysis (PCA), to identify the most relevant to include in the final model of iDNA richness. This showed that sites cluster based on habitat type, and that mean VPD and mean temperature showed the greatest orthogonal difference (Figure S3). Thus, to distinguish between warm/cool and wet/dry sites, we continued the analysis using these two mean microclimate variables. We tested two response variables, alpha diversity using a Poisson generalized linear model (GLM), and the exponent of Shannon's diversity index (Hill number where *q* = 1) with a normal distribution. We included mean temperature and mean VPD as continuous fixed effects, and habitat type with three levels (old growth, twice logged and heavily logged) as a categorical effect. As the number of leeches collected per site varied, we included this variable as an offset term in all models, which specifies the amount of variation in the response variable that can be attributed to the offset term (Crawley, 2007), such as the number of leeches. This method sets the regression coefficient to 1 and allows the diversity of a sample to be calculated given the number of leeches sequenced. As a null model for comparison, we removed all terms except the offset.

Using an Akaike information criterion (AIC) approach, we generated every potential model, from the full to the null model, and calculated the ∆AIC values and AIC weight. We then removed models which made up less than 5% of the cumulative weight. To summarize the relative importance of each variable, we calculated a weighted proportion of best fitting models which retained the variable of interest, as the cumulative AIC weight divided by the maximum AIC weight of the model set. All model analyses were conducted in base r and with “bbmle” (Bolker, 2014) and “broom” (Robinson & Hayes, 2020), and the PCA and figure (Figure S3) were generated using base r (R Core Team, 2019) and “factoextra” (Kassambara & Mundt, 2020).

## 3 RESULTS

### 3.1 Sequence filtering and taxonomic identification

Following filtering steps, all samples above a genus level were removed with the exception of a single hit to the family Felidae, which occurred as a high copy number. Because there are multiple felid genera in Borneo, we retained the family‐level designation. Additionally, a large number of unique sequences (296 of prefiltered 1,590) were assigned to the genus *Sus*. Although domestic pig (*Sus scrofa*) DNA can often be a source of laboratory contamination in eDNA studies, only 93 of these 296 were assigned directly to *S. scrofa* by obitools and removed. Given that *S. scrofa* does not occur naturally in Borneo, is not farmed in the study area and did not occur in the PCR‐negative controls, we assigned the remaining 203 unique sequences to the only native pig species, Bornean bearded pig (*Sus barbatus*). This taxon is the most abundant large‐bodied mammal at the SAFE study site, and is commonly recorded in large groups by camera traps (Deere et al., 2017).

After comparing the filtered reads to the ecopcr database we identified 1,362 unique sequences. We collapsed these by taxon and leech pool, resulting in 92 detections across 57 leech pools with an average of 1.6 detections per pool (range = 1–5, median = 1, Table 1). The mean number of individual leeches per pool was 9.8 (range = 5–11, median = 10). The detections were assigned to 10 mammal families (Table 2), which could further be identified as belonging to 14 genera (with the exception of the Felidae hit). Of these 14, nine could be confidently assigned to the species level based on the knowledge of a single species known to occur within Sabah. An additional four genera each contain two species that co‐occur on Borneo, and were thus only assigned to genus. These taxa, along with their common names, are: *Macaca fascicularis* (long‐tailed macaque) and *Macaca nemestrina* (southern pig tailed macaque), *Muntiacus muntjac* (common southern red muntjac) and *Muntiacus atherodes* (Bornean yellow muntjac), *Tragulus napu* (greater mousedeer) and *T. kanchil* (lesser mousedeer), and *Hystrix brachyura* (Malay porcupine) and *Hystrix crassispinis* (thick‐spined porcupine).

**TABLE 2:** Taxonomic assignments to order‐, family‐, genus‐ and, where possible, species‐level identities

Order	Family	Genus	Species	Common name	Occurrences	Sites
Cetartiodactyla						
	Cervidae	*Muntiacus*		Muntjac	8	6
		*Rusa*	*Rusa unicolor*	Sambar deer	21	9
	Suidae	*Sus*	*Sus barbatus*	Bearded pig	24	8
	Tragulidae	*Tragulus*		Mouse deer	6	2
Carnivora						
	Felidae*			Asian wild cat species	1	1
	Ursidae	*Helarctos*	*Helarctos malayanus*	Sun bear	2	1
	Viverridae	*Arctogalidia*	*Arctogalidia trivirgata*	Small toothed palm civet	1	1
		*Hemigalus*	*Hemigalus derbyanus*	Banded civet	1	1
		*Paguma*	*Paguma larvata*	Masked palm civet	1	1
		*Viverra*	*Viverra tangalunga*	Malay civet	5	4
Pholidota						
	Manidae	*Manis*	*Manis javanica*	Sunda pangolin	1	1
Primate						
	Cercopithecidae	*Macaca*		Macaque	2	2
						
Proboscidea						
	Elephantidae	*Elephas*	*Elephas maximus*	Elephant	1	1
Rodentia						
	Hystricidae	*Hystrix*		Porcupine	16	6
		*Trichys*	*Trichys fasciculata*	Long‐tailed porcupine	2	2

Note

All assignments could be made to at least genus apart from one, an unknown Felidae, which is indicated by an asterisk. The number of occurrences of each assignment and the number of sites where it was found are given.

### 3.2 Accumulation of diversity across habitat types

**FIGURE 2:**
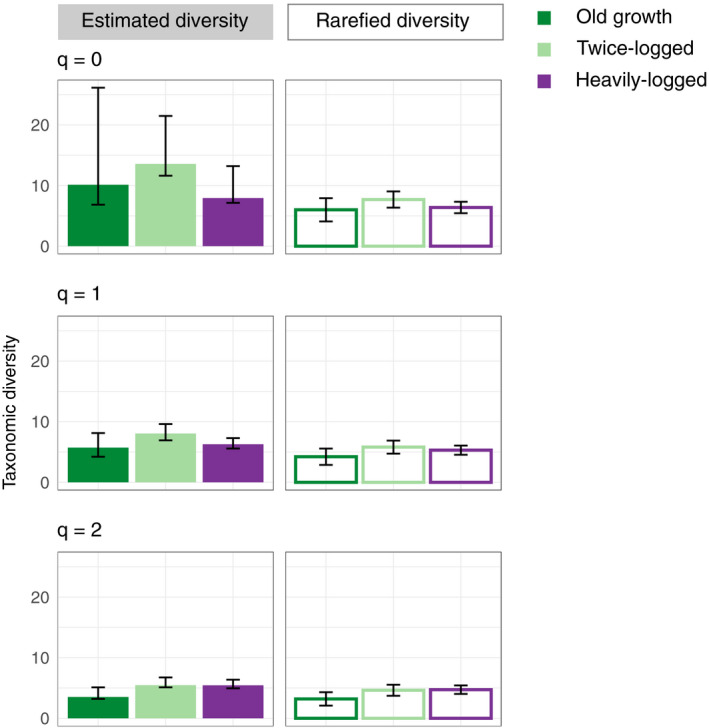
Plot of the estimated asymptotic diversity estimates (solid bars) and the rarefied diversity values (open bars) for diversity where *q* = 0, 1, 2, for each of the habitat types. The samples are rarefied to the smallest sample, which is for the old growth habitat (*n* = 12). Error bars shown are the 95% confidence intervals

Twice logged forest sites had a greater estimated diversity where *q* = 0 (species richness) compared to the heavily logged forest, but this overlapped with the old growth habitat. Where *q* = 2, both logged habitat types had higher estimates of diversity than the old growth forest (Figure 2). When sample sizes were rarefied to the smallest sample size, the old growth habitat had the lowest diversity estimates at all three values of *q*, but the confidence intervals all overlapped (Figure 2).

**FIGURE 3:**
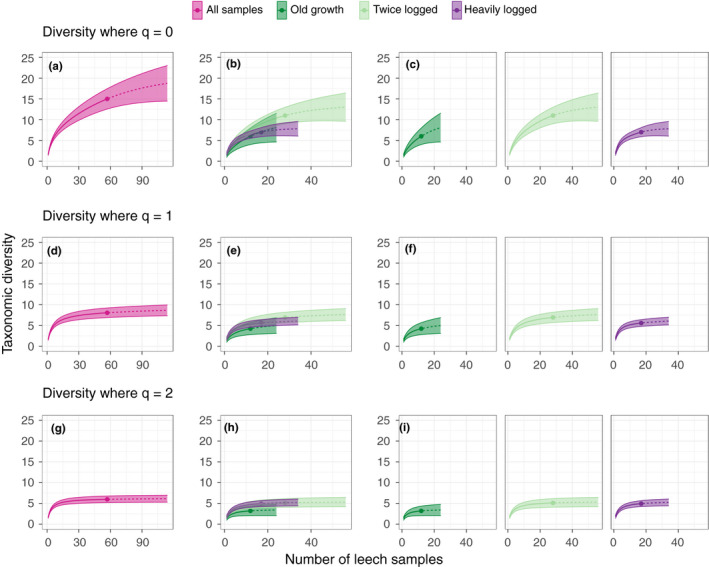
Diversity accumulation curves at the genus level comparing the effect of increasing leech samples on the detected diversity within different habitat types. Curves are calculated using three orders of Hill numbers, *q* = 0, 1 and 2—equivalent to species richness, Shannon diversity index and Simpson index, respectively, and presented with 84% confidence intervals. The solid line represents the rarefied values, and the dashed line represents the extrapolated values and is extended to double the reference sample (empirical value, solid circle), following Chao et al. (2014). Panels (a), (d) and (g) show the diversity accumulation of all samples from all habitats. Panels (c), (f) and (i) are curves of the same data as (b), (e) and (h) but separated for clarity

When *q* = 0, twice logged forest showed more rapid accumulation of mammal genera compared to the heavily logged forest, but values fell within the CIs of the curve for old growth forest (Figure 3b). All habitat types overlapped at *q* = 1 although old growth forest was lower than the other habitats (Figure 3e), whereas at *q* = 2 the former habitats overlapped with each other but did not overlap with the lower accumulation curve of the old growth (Figure 3h). This may indicate that the observed differences between the two degraded habitats are driven by unevenness. The curves for all three habitats approached an asymptote where *q* = 1 and 2 (Figure 3d, e, g, h).

### 3.3 Changes in community composition across the landscape

**FIGURE 4:**
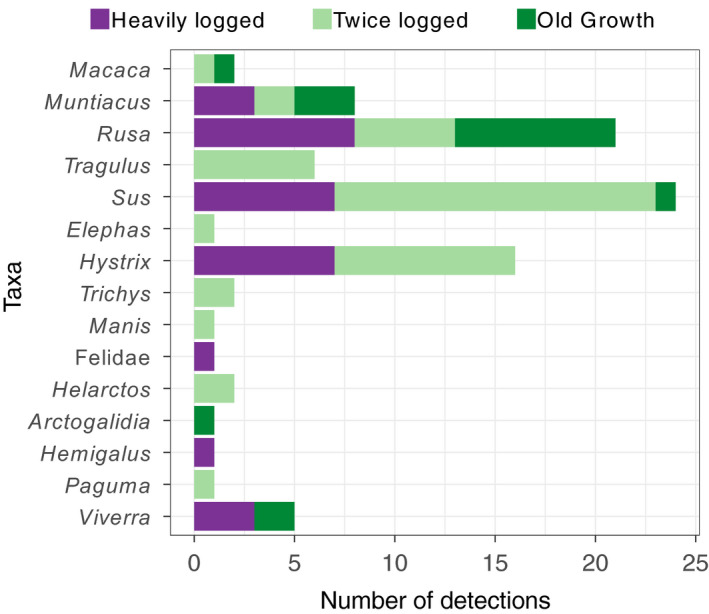
The number of detections of each genus across the three different habitat types. Felidae is the only assignment at family level

Only three taxa were not detected in the twice logged forest: small toothed palm civet (*Arctogalidia trivirgata*), banded civet (*Hemigalus derbyanus*) and the unidentified felid species (Figure 4). Bearded pigs, muntjacs and sambar deer were detected in all habitat types. The most diverse family detected was the Viverridae with four different genera recorded, and overall the most abundant detections were of bearded pig (*S. barbatus*), followed by sambar deer (*Rusa unicolor*). Six of the taxa were rarely detected, with a single occurrence (including species of conservation concern as designated by the IUCN Red List; IUCN, 2020): Asian elephant (*Elephas maximus*, Endangered), Sunda pangolin (*Manis javanica*, Critically Endangered) and masked palm civet (*Paguma larvata*, Least Concern), all of which were detected only in the twice logged forest, and the small‐toothed palm civet (*A. trivirgata,* Least Concern) which was detected only once in the old growth forest (Figure 4). There was a single detection of the banded palm civet (*H. derbyanus*) which is listed as Near Threatened (NT) by the IUCN red list in heavily logged forest.

**FIGURE 5:**
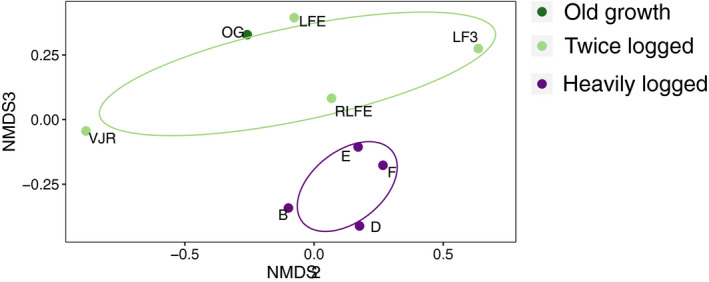
Nonmetric multidimensional scaling (NMDS) ordination based on genera presence/absence using the Chao dissimilarity index showing the difference in community structure between sampling sites. The second and third axes are displayed. Ellipses show the 95% confidence area around the habitat type group centroids

The NMDS ordination based on the Chao dissimilarity coefficients converged with a stress value of 0.1 on two axes (*k* = 2) and 0.04 on three axes (*k* = 3). Visualizing axis 2 versus axis 3 revealed a clear separation of communities detected in twice logged and heavily logged forest (Figure 5). The PERMANOVA test showed a significant effect of habitat type (*df* = 2/56, *R* ^2^ = 0.12, *F* = 3.81, *p* < 0.01).

### 3.4 Effects of habitat and microclimate on mammal diversity

Of all the richness models (*n* = 8, no interactions considered), six models had a cumulative weight of > 0.95. The null model and the mean temperature model accounted for over half of the cumulative weight of the final model set (Table 3). Mean temperature was also retained in a higher proportion of the total models compared to the other variables (mean temperature = 0.35, mean VPD = 0.23, habitat = 0.15). However, no model can be considered a better fit, or no term can be considered to have greater importance, given that the null model (including the offset term only) was retained in the final set of models. Therefore, the rate of detection appears to be driven mainly by the number of leeches collected.

**TABLE 3:** Model comparison for models which are within 0.95% of the cumulative AIC weight of the total model set, for two diversity metrics, *q* = 0 and *q* = 2

Model	∆AIC	AIC*w*
Diversity order of *q* = 0		
Null	0	0.40
Mean temperature (0.35)	1.47	0.59
Mean VPD (0.23)	1.99	0.73
Habitat (0.15)	3.37	0.81
Mean temperature + mean VPD	3.40	0.88
Mean temperature + habitat	3.64	0.94
Diversity order of *q* = 1		
Mean temperature + habitat	0	0.28
Null	0.56	0.49
Mean temperature (0.63)	1.18	0.64
Mean temperature + mean VPD + habitat	1.79	0.76
Mean VPD (0.26)	2.56	0.83
Habitat (0.47)	2.94	0.90
Mean temperature + mean VPD	3.04	0.958*

Note

Models are in order of descending ∆AIC for each order of *q*. AIC*w* is the cumulative AIC weight of the models. The weighted proportion of retention for each of the main terms is given in parentheses. This is the sum of the cumulative weight of each model in which the term appears, divided by the maximum weight, as a summary of its relative importance. All models include the offset term of total number of leeches per pool. The final model (*) for *q* = 1 is included as the last model over 0.95 cumulative weight.

When comparing community diversity using the Hill number where *q* = 1, we found that the model with the lowest AIC was the one that included temperature and habitat type, followed by the null model with only the offset term included. These two models account for almost half of the cumulative weight = 0.49 (Table 3). Mean temperature had the highest proportion of retention in the models (0.63), followed by habitat (0.47) and then mean VPD (0.26). As with the richness models, because the null model has been retained as one of the models within the AIC (Table 3), this indicates that no conclusions can be drawn on the influence of the variables in the model.

## 4 DISCUSSION

We show that iDNA‐based surveys can successfully identify differences in mammal communities in forest habitats of varying quality. From our 57 pooled samples of 557 leeches, we identified at least 14 mammalian genera, representing 10 families. The highest abundance of detections was from large ungulates, specifically the bearded pig, sambar deer and muntjac (order Cetartiodactyla), followed by detections of the genus *Hystrix*, representing either the Malay porcupine (*Hystrix brachyura*) or the thick‐spined porcupine (*Hystrix crassispinis*). Camera trapping studies at other sites in Sabah have also recorded these taxa at high abundances (Bernard et al., 2013, 2014). The highest number of genera (*n* = 4) for a single family was detected for the Viverridae (order Carnivora), representing half of the species diversity from this taxon in Sabah (Payne & Francis, 1985). In addition to these detections of common species, we also detected a number of less commonly recorded species, including the pangolin and sun bear.

The importance of leeches in the detection of rare species is supported by a recent study that combined camera trapping and iDNA sampling in Vietnam, where leeches increased detection records for rare species (marbled cat, Owston's civet and Asian black bear) compared to cameras alone (Tilker et al., 2020). Although we did not record a complete catalogue of mammals known to occupy our study area, we did detect the majority of large‐bodied, nonvolant mammals. However, we only detected one genus of diurnal primate (macaque), despite the fact that several occur in the area. The presence of macaques in our sample might reflect the fact that they spend more time on the ground compared with gibbons, leaf‐monkeys and the orangutan (Hanya et al., 2020). With the exception of the porcupines, we recorded no rodents or bats, which together account for the largest components of mammalian biodiversity in Bornean rainforests. This trapping bias illustrates the limitation of leeches as iDNA samplers, and probably reflects their foraging behaviour on the ground or in the understorey. Thus, combining leeches with other invertebrate samplers, or with complementary trapping methods, may help to obtain more complete taxonomic coverage (i.e., dipteran flies, Gogarten et al., 2019; Hoffmann et al., 2018).

### 4.1 Habitat differences in mammalian diversity

When looking at leech pools from all habitats, the accumulation of genera approached an asymptote for higher orders of *q*, equivalent to Shannon's diversity index and the Simpson index. However, for *q* = 0 (species richness), the curve did not reach a plateau, indicating that more sampling is needed. This under‐sampling appears to be driven by the old growth habitat, for which actual species richness is expected to be much higher. Interestingly, we found some evidence that samples from the old growth forest contained lower levels of taxonomic diversity compared to the other two habitat types. This finding may reflect elevated numbers of generalist species in degraded habitats, and broadly agrees with previous findings. Wearn et al. (2017), for example, used camera trap data to characterize and compare diversity of Bornean mammal guilds between old growth and logged forest, and found consistently higher levels of diversity in the latter. Despite this, the lower sampling effort in the old growth sites in our study means that any conclusions regarding this habitat type must be considered tentative.

Within the two categories of degraded forest, we found higher alpha diversity as measured by species richness in the twice logged forest than in the more heavily logged forest. At higher orders of *q*, however, we found much greater overlap between these two habitats. Overall, the accumulation of diversity was seen to reach an asymptote at *q* = 2, consistent with the expectation that the reciprocal of the Simpson index is suited to cases of near‐complete sampling. Taken together, these results imply that the differences in alpha diversity between forests with different logging histories are probably driven by small numbers of uncommon species, and that when the number and evenness of detections is considered (i.e., higher orders of *q*), then the overall diversity becomes more similar between these habitats. Thus, the use of higher orders of *q* appear to be less sensitive to biases from sampling effects due to the fact they are dominated by the incidence of commonly occurring species.

Multiple previous studies from Borneo have shown the importance of logged forests within modified landscapes for supporting biodiversity. Indeed, once‐logged forests appear to retain much of the mammal species richness of primary forests (Putz et al., 2012), although subsequent analyses have revealed effects on community composition (Costantini et al., 2016; Gray et al., 2014; Wearn et al., 2017). Here we show that iDNA‐based sampling can recover differences in community composition, with species of conservation concern (e.g. pangolin) only recorded in the better quality habitat. However, unlike these earlier studies, we were unable to test for species richness declines in plantations and/or pastures, due to the absence of leeches in these relatively arid environments, which also represent the end points of forest degradation (e.g. Edwards et al., 2014).

### 4.2 Effect of microclimate on detection of diversity

Our models of richness and Shannon's diversity index showed no clear effect of microclimate. In all cases, the null model, which only includes the total number of leeches collected as an offset, was retained in the final set of models, and therefore cannot be excluded as the best description of the data. This result suggests there is a strong effect of leech abundance, and that this drives subsequent numbers of detections. Thus, if microclimate‐mediated responses do drive differences across habitats, they appear to mainly affect leech density rather than either their feeding preferences or success. Nonetheless, a lack of clear effect of temperature and humidity was somewhat surprising given the mounting evidence that microclimate and microrefugia play important roles in tropical ecosystems (Hardwick et al., 2015; Jucker et al., 2018; Senior et al., 2018). However, we cannot completely rule out an effect of temperature on detection given that this variable showed the highest proportion of retention across models of species richness. Ultimately, we suggest that microclimate effects on iDNA‐based detections need to be tested in a larger dataset that includes better coverage of old growth habitat.

### 4.3 Other considerations for iDNA studies

With increasing numbers of studies using invertebrate samplers to assay vertebrate diversity, there is a growing appreciation of the importance of several aspects of study design, especially with respect to the choice of sampler, laboratory procedures and statistical frameworks. First, the choice of sample should not only consider detection biases introduced by habitat or microclimate but should also consider potential species differences. Previously, for example, we showed that the tiger leech, *Haemadipsa picta*, yielded more mammal diversity than did the brown leech, *Haemadipsa zeylanica*, probably as a result of the former species’ greater tendency for arboreal foraging, and wider distribution (Drinkwater et al., 2018). This species difference in detection probability has also been supported by Abrams et al. (2019). Most iDNA studies to date have also treated leeches and other invertebrates as passive samplers, whereas these taxa may exhibit some degree of active prey choice. Evidence for prey selection was previously suggested for the Japanese blood feeding leech (*Haemadipsa japonica*), where mammals seen on camera traps differed from those detected by iDNA (Hanya et al., 2019). Here the authors concluded that, due to apparent nonpassive foraging, this leech species might be a poor choice of sampler for generating a comprehensive biodiversity inventory (Hanya et al., 2019). More work is needed to determine if Bornean leeches also exhibit active prey choice.

Regarding laboratory procedures, rates of detection in iDNA studies will be heavily influenced by methodological choices, which in turn will depend on the research question (Alberdi et al., 2017). For example, the use of pooling to increase throughput is particularly useful in large cross‐continental studies (e.g. Schnell et al., 2018; Tessler et al., 2018) whereas analysing individual leeches separately has been employed in site specific studies where resolution is more important (e.g. Schnell, Thomsen, Wilkinson, Rasmussen, et al., 2012; Weiskopf et al., 2017). In our study, pooling individual leech DNA extracts together increased the scale of our analysis, yet at the same time prevented us from relating mammals to the exact location and timing of individual leech captures. This may be an important factor in single species‐driven studies, or in the detection of rare species, the most famous example being the development of leech‐based surveys for the detection of the saola, *Pseudoryx nghetinhensis,* in Vietnam (The Saola Working Group, 2020; WWF, 2013). The common approach of pooling samples may also lead to the masking of DNA templates of rare species by those of common species, such as the bearded pig in our study (also see Pompanon et al., 2012).

An additional methodological consideration that is likely to have an important impact on detections is the choice and number of DNA markers. Although the 16S marker used in our study commonly features in iDNA work, it cannot resolve among some closely related taxa, such as congeneric species within the family Felidae. Thus, additional markers would almost certainly allow us to resolve more species within our sample. For example, in another recent iDNA‐based survey of mammals conducted in Sabah, Abrams et al. (2019) recorded just three additional genera (*n* = 18) from nearly double the number of leeches (*n* = 1,532); however, their use of three mitochondrial markers (16S, 12S and *cytB*) allowed them to identify 22 species (Abrams et al., 2019). Thus, where possible, future studies should aim to use multiple markers to increase taxonomic resolution, as also recommended by Axtner et al. (2019), who proposed a new laboratory workflow that included the use of three mitochondrial markers alongside multiple replicates.

Finally, measures of diversity and habitat effects from iDNA‐based monitoring will be influenced by statistical approaches, including the choice of diversity metrics and models, for example Hill numbers (Chao et al., 2014). This framework has also been developed specifically for molecular data from OTU‐based metabarcoding studies (Alberdi & Gilbert, 2019). Recently, statistical frameworks that account for imperfect detections, notably occupancy models, have also been applied to eDNA data (Dorazio & Erickson, 2018; Griffin et al., 2019; Hunter et al., 2015; Schmidt et al., 2013). Abrams et al., (2019) applied single‐season occupancy models to leech iDNA and camera trap data, and found comparable probabilities for mammals across these techniques. The incorporation of occupancy‐based methods has also been applied in the context of poaching and defaunation for identifying priority conservation areas in Vietnam, again demonstrating the power of a combined approach (Tilker et al., 2020). Furthermore, Broms et al. (2015) have developed an extension of the occupancy modelling framework which utilizes Hill numbers of diversity.

In our study, by using pooled samples of a single leech species to generate Hill numbers of taxonomic diversity, we find differences across forest types with different logging histories. Therefore, although leeches cannot provide an exhaustive catalogue of the mammals present at a given site, leech iDNA is nevertheless still capable of assaying a representative mammalian community that includes rare species. Advances in methods and sequencing technologies will be needed to further enhance accuracy and confidence in iDNA detections. Additionally, gaining a deeper understanding of leech ecology and integrating more sophisticated models will be key to the wider uptake of such methods in practice. Nevertheless, our findings showcase the potential for using iDNA‐based sampling methods for biodiversity surveys in degraded and pristine tropical forests.

## AUTHOR CONTRIBUTIONS

R.D. and S.J.R. designed the research; R.D. conducted the fieldwork with logistical help from H.B. and E.M.S.; R.D. conducted the laboratory work with input and resources from M.T.G., E.L.C. and S.J.R. O.T.L., E.M.S., M.J.S. and S.J.R. secured funding for the project. T.J. provided the microclimate data and helped with analyses; D.A.C., T.J. and T.S. helped with LiDAR data and interpretation; R.D. analysed the data with bioinformatics input from J.H.T.; R.D. and S.J.R. wrote the paper with input from all co‐authors.

## Supporting information

Fig S1‐3

Table S1‐2

## Data Availability

Data are available on the SAFE project Zenodo repository (http://doi.org/10.5281/zenodo.4095374) (Drinkwater et al., 2020). Raw sequence data are available on the NCBI short read archive, with the SRA BioProject accession no: PRJNA672059 (https://www.ncbi.nlm.nih.gov/sra/PRJNA672059).
