# Supplementary material for: Leech blood‐meal invertebrate‐derived DNA reveals differences in Bornean mammal diversity across habitats
Source: Mol Ecol. 2020 Nov 27;30(13):3299–312. doi: 10.1111/mec.15724 (PMC8359290; doi:10.1111/mec.15724)
Supplement: Supplementary file 1 — Fig S1‐3 [file MEC-30-3299-s001.docx]

**Supplementary Material -**

**Leech blood-meal iDNA reveals differences in Bornean mammal diversity across habitats**

Drinkwater, R^1^, Jucker, T.^2^, Potter, J. H. T.^1^, Swinfield, T.^3^, Coomes, D. A.^3^ , Slade, E. M.^4,5^, Gilbert, M. T. P.^6,7^, Lewis, O. T.^4^, Bernard, H.^8^, Struebig, M. J.^9^, Clare, E. L.^1^ & Rossiter, S. J.^1^


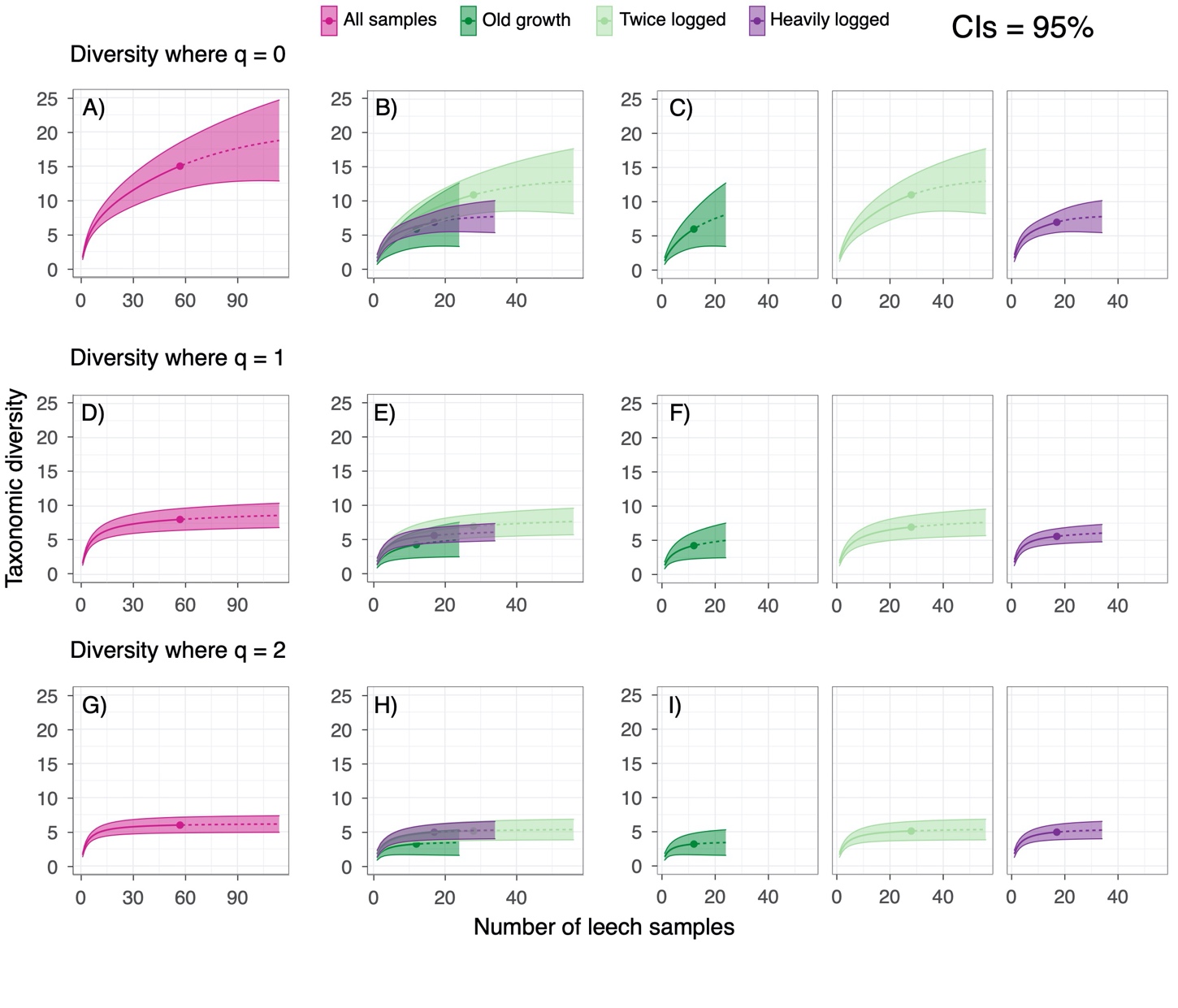


**Supplementary Figure S1.** Diversity accumulation curves at the genus level comparing the effect of increasing leech samples on the detected diversity within different habitat types. Curves are calculated using three orders of hill numbers, q = 0, 1 & 2 - equivalent to species richness, Shannon diversity index and Simpson index, respectively and presented with 95% confidence intervals. The solid line represents the rarefied values, and the dashed line represents the extrapolated values and is extended to double the reference sample (empirical value, solid circle), following Chao et al (2014). Panels A, D & G show the diversity accumulation of all samples from all habitats. Panels C, F & I are curves of the same data as B, E & H but separated for clarity.

**Supplementary Figure S2.** Boxplots showing the variation in microclimate metrics across the sites where leeches were sampled. A) The maximum temperature, B) mean temperature, C) maximum vapour pressure deficit (VPD), and D) mean VPD. Values are calculated from data used in Jucker et al (2018). The black dots show the individual values measured at the plot level.

**Supplementary Figure S3**. Principal components analysis (PCA) biplot of microclimatic variables (in yellow) measured at all the plots in the three habitat types; old growth sites at DVCA (dark green circles), twice logged (light green triangles) and heavily logged (purple squares) at the SAFE project. The percent variation of the first two axes are shown – PCA1 = 77.7% and PCA2 = 19%.
